# "It’s Not Smooth Sailing": Bridging the Gap Between Methods and Content Expertise in Public Health Guideline Development

**DOI:** 10.15171/ijhpm.2019.137

**Published:** 2020-01-01

**Authors:** Nicholas Chartres, Quinn Grundy, Lisa M. Parker, Lisa A. Bero

**Affiliations:** ^1^Charles Perkins Centre, Faculty of Medicine and Health, School of Pharmacy, The University of Sydney, Sydney, NSW, Australia.; ^2^Faculty of Nursing, University of Toronto, Toronto, ON, Canada.

**Keywords:** Guidelines, Guideline Development, Public Health, Methods, Australia

## Abstract

**Background:** The development of reliable, high quality health-related guidelines depends on explicit and transparent processes, methods aimed at minimising risks of bias and the inclusion of all relevant expertise and perspectives. While the methodological aspects of guidelines have been a focus to improve their quality, less is known about the social processes involved, for example, how guideline group members interact and communicate with one another, and how the evidence is considered in informing recommendations. With this in in mind, we aimed to empirically examine the perspectives and experiences of the key participants involved in developing public health guidelines for the Australian National Health and Medical Research Council (NHMRC).

**Design:** This study was conducted using constructivist grounded theory as described by Charmaz, which informed our sampling, data collection, coding and analysis of interviews with key participants involved in developing public health guidelines.

**Setting:** Australian public health guidelines commissioned by the NHMRC.

**Participants:** Twenty experts that were involved in Australian NHMRC public health guideline development, including working committee members with content topic expertise (n=16) and members of evidence review groups responsible for evaluating the evidence (n=4).

**Results:** Public health guideline development in Australia is a divided process. The division is driven by 3 related factors: the divergent disciplinary background and expertise that each group brings to the process; the methodological limitations of the framework, inherited from clinical medicine, that is used to assess the evidence; and barriers to communication between content experts and evidence reviewers around respective roles and methodological limitations.

**Conclusion:** Our findings suggest several improvements for a more functional and unified guideline development process: greater education of the working committee on the methodological process employed to evaluate evidence, improved communication on the role of the evidence review groups and better facilitation of the process so that the evidence review groups feel their contribution is valued.

## Background

Due to the large number of organisations and governments that produce guidelines, end-users are often presented with contradictory recommendations and guidelines of varying quality.^1,2^ In order to improve the quality of guidelines, several organisations around the world such as the World Health Organization (WHO),^3^ the National Institute for Health and Care Excellence,^4^ and the United States Community Preventive Services Task Force^5^ have developed standards and criteria for their development. For example, *the WHO Handbook for Guideline Development* , 2nd Edition requires that the process for developing a recommendation is explicit, transparent, and uses methods aimed to minimise risk of bias; the guideline development group includes all relevant expertise and perspectives, and that recommendations consider benefits and harms as well as other relevant factors.^3^ Currently in Australia, there are standards for guidelines approved by the National Health and Medical Research Council (NHMRC) and guidance to achieve these standards is being developed.^6,7^

GRADE (The Grading of Recommendations Assessment, Development and Evaluation) is widely endorsed as a methodology for clinical guideline development worldwide, including by the WHO and NHMRC.^3^ GRADE allows for a transparent rating of the quality of evidence and rates the confidence in effect estimates for benefits and harms as high, moderate, low, or very low.^8^ GRADE has been optimised for evaluating clinical interventions and randomised controlled trials.

Public health guidelines offer recommendations to prevent ill health or to improve the health of a population, which are tailored to a specific audience (ie, public health policy-makers, healthcare providers, patients, caregivers, the public and other relevant stakeholders).^3,9^ For example, NHMRC guidelines assess the health harms of living near a windfarm^10^ or provide dietary advice for Australians.^11^ Unlike clinical practice guidelines, the available evidence for the development of public health guidelines is seldom from randomised controlled trials but instead is often derived from observational studies such as cohort studies, case controls, or time series analyses. Further, GRADE has not been developed to account for all important considerations related to public health guideline development, for example it does not provide explicit guidance for when evidence is linked across a causal pathway.^12^ Depending on the risk of bias tool used, GRADE can also downgrade non-randomised controlled trial evidence, even if this is the only and most appropriate type of evidence available. The use of GRADE for developing public health guidelines or conducting systematic reviews in the field of public health has been previously studied.^13^ While it has been recognised as a systematic and transparent process of evaluating the evidence, challenges have been identified in its use due to the complex nature of public health exposures.

Although the most rigorous and trustworthy guidelines are evidence-based, evidence may not be the greatest influence in the formation of guidelines.^14^ In clinical practice guideline development, previous experiences and beliefs that are not consistent with the research evidence are prioritised when developing recommendations.^15^ Further, the status and, therefore, power of the guideline development groups have been shown to influence the level of input made by individuals in multidisciplinary guideline panels.^16^

Several studies have examined how participants involved in public health guidelines translate evidence into recommendations. Guideline development group members may conceptualise the guideline development task differently, with some prioritising the evidence in informing their decision-making, while others, their disciplinary expertise.^17^ Although the diversity of opinions in such groups brought tensions, diversity was vital in making informed judgements, relevant to making recommendations.^17,18^ Tensions have also been experienced between guideline development groups and those conducting the evidence synthesis.^19^

While the methodological aspects of the guideline process have been a focus for improving the quality of guidelines,^3-5^ less is known about the guideline development groups social processes. We conducted a grounded theory study to understand the perspectives and experiences of those involved in the development of public health guidelines. Consistent with a grounded theory approach, we define social processes as participants’ actions, interactions, and decision-making within the context of a guideline development process with a specified beginning and endpoint.^20^ We aimed to understand the perspectives and experiences of the 2 key groups of participants involved in developing public health guidelines for the NHMRC: the evidence review groups and the working committees (see Table 1 for a description of these roles). We included these 2 groups to allow a broad understanding of the guideline process, to learn about the relationships these groups of participants have with one another, and how, if at all, these relationships shape the guideline process. By understanding these viewpoints, we aimed to gain a greater understanding of the social influences on the guideline development process, including, how guideline group members interact and communicate with one another, and how the evidence is considered in informing recommendations. Such influences on the guideline development process are not apparent in the various handbooks written on the methodological procedures and technical aspects of guideline development.

**Table 1:** Roles and Responsibilities of the Working Committee^a^ (Verbatim Description From NHMRC Guidelines for Guidelines Website)^21^ and Evidence Review Group

**Group Member**	**Key Responsibilities**
All members	• Agree on the scope, questions and P[I/E]CO • Contribute constructively to meetings, including approving the minutes • Declare all relevant interests so that conflicts of interest can be identified and managed • Develop actionable recommendations based on reviews of evidence • Identify potential implementation issues and propose steps to overcome them • Assess the acceptability and feasibility of the recommendations • Weigh the potential risks and benefits of treatment • Make decisions on what information should be included • Consider and deliberate on public consultation submissions
Chair	• Contribute to the drafting of terms of reference and formation of the guideline development group • Facilitate group processes and promote balanced participation of group members • Support effective consumer involvement • Manage conflicts of interest during meetings • Ensure that the group stays focused and task oriented • Delegate work and co-ordinate output of the group
Content experts	• Apply their knowledge to improving the identification of relevant evidence • Advise on how to identify best practice in areas for which limited evidence is available • Identify, critically appraise and synthesise evidence into a format useful for developing recommendations^b^ • Assist the group in understanding the evidence and evidence-to-decision process^b^
Consumers	• Consider to what extent published evidence reflects outcome measures that consumers consider important • Ensure that questions and recommendations address consumer issues and concerns • Ensure that the guideline is worded appropriately
Methodological experts^c^	• Identify, critically appraise and synthesise evidence into a format useful for developing recommendations • Assist the group in understanding the evidence and evidence-to-decision process • Maintain comprehensive records
Evidence review group^d^	• Conduct an independent evidence evaluation of all the relevant scientific research, using internationally recognised systematic review methods to perform the evidence evaluation to the highest possible standard • Assist the guideline working committee to understand the evidence evaluations

Abbreviations: P[I/E]CO; Participant, Intervention/Exposure, Comparator, Outcome; NHMRC, National Health and Medical Research Council. ^a^ In the current NHMRC Guideline for Guidelines website from which we sourced this table, the working committees we refer to are called ‘guideline development groups.’ However, for consistency with how they are described in our study we have called them working committees. ^b^ This is often considered a core task of content experts, particularly in the absence of a methodological expert. ^c^ There are methodologists on the working committee, but they do not complete the reviews as described here. ^d^ Not verbatim text. We included this description for our study only.

## Methods

This study was conducted using constructivist grounded theory as described by Charmaz, which informed our sampling, data collection, coding and analysis.^20^ This type of grounded theory asserts that the knowledge produced is contextually created by the participants of the research and by the researcher. Grounded theory was explicitly developed to analyse social processes that include participants’ actions and interactions and how they are shaped by their diverse and complex experiences.^20,22^ We sought to learn about the various unseen situations, relationships and tacit networks in the guideline process, seeking to unearth any relationships of power and communication that are not captured in the published guideline documents.^22^ We remained aware of our professional and disciplinary expertise and kept coding and interpretation close to the data.

### Participants and Sampling

We selected participants that were part of the development of a public health guideline published from 2013 or later, or currently under development for the NHMRC in Australia.

We use the term ‘guideline’ in this study to represent the guidelines, information papers and statements that the included groups produced for the NHMRC. We use the term ‘working committee’ in this study to represent those participants that were part of working committees, reference groups and advisory groups that reported to the Council of the NHMRC (Table 1).We use the term ‘evidence review groups’ to represent those participants that were contracted by NHMRC to review and evaluate the evidence for targeted questions that informed the development of a ‘guideline’ (Table 1).

Initially we sampled purposively to seek multiple perspectives from those with in-depth and varied experiences with the guideline development process. We reasoned that the perspectives and opinions of the process would vary between guidelines and the roles that were undertaken as described in Table 1. We therefore invited all members of guideline working committees and evidence review groups to be part of the study. As we used constant comparative analysis techniques throughout the process, after our initial data collection and analysis, we used theoretical sampling to seek data that would continue to develop and refine our emerging theoretical concepts.^20^ That is, we modified our recruitment strategy in response to what we had heard from early participants in order to fill gaps in our data and to test and advance our developing ideas

We identified participants by emailing the NHMRC contact addresses for randomly selected public health guideline topics on the NHMRC websites and asked the NHMRC contact person to invite all participants that had been involved in the development of the guideline. We provided a participant information statement to share with eligible participants. If a guideline participant expressed interest in being involved in the study, we were given their contact information by the NHMRC. We also contacted potential participants suggested by colleagues or other participants that we interviewed. All information necessary to contact these participants was in the public domain.

### Data Collection

Between April 2018 to July 2018, in-depth, semi-structured interviews were conducted by NC face to face in the participant’s or our research team workplace, or over the telephone when participants were unavailable to meet in person. Both face to face and interviews conducted over the phone were of similar nature and length^23^ (39-77 minutes; average 57 minutes). The interview guide was designed to evoke the participants’ opinions and experiences in being involved in the guideline development process and to remain open to what participants’ deemed salient to the process of guideline development (see Table 2). The interviews were digitally recorded and professionally transcribed. Every participant in the study gave written or verbal consent and all were informed that they were free to withdraw from the study at any stage during the process.

**Table 2:** Interview Guide

**Question**	**Prompt**
Tell me a little about your role in the guideline development?	Specific responsibilities? Level of responsibility? Level of input?
Tell me about the guideline development process?	How was evidence defined? How was the evidence summarised? How was the quality of the evidence assessed, if at all? How was evidence synthesised? What other factors contributed to rating the quality of the overall body of evidence? What factors (other than the evidence reviewed) contributed to the rating of recommendations?
If any, what do you feel were the key challenges in the process?	What was done in the absence of evidence? Were there challenges in assessing the risks of bias or quality of the evidence? Was a formal method applied for rating recommendations eg, GRADE? If so, what worked or did not work about this method?
What else should I know about the process?	Stakeholder input? Relationships between various experts? Lack of standardised criteria?

Abbreviation: GRADE; Grading of Recommendations Assessment, Development and Evaluation.

### Analysis

NC wrote field notes immediately after each interview to capture thoughts on the interview, participants and initial ideas. Transcripts were analysed as soon as they were received. We used initial line by line coding to inductively generate multiple ideas from our early interviews and data.^20^ We identified a group of codes that captured the relationships between the various groups of participants and their views on the methodological challenges of the guideline development process.

Throughout the study NC wrote case based and conceptual memos, which were discussed with the authorship team during regular meetings.^20^ These memos drew on the emerging data and were used to write the initial codes and to develop our thinking on participant views about the guideline decision-making processes, including identifying similarities and difference between the various groups of guideline participants. This early analysis influenced questions for future interviews, as we sought to test emerging concepts from the data and refine our developing ideas.

### Reflexivity

The authors bring different expertise and methodological perspectives to this study; LB is primarily a quantitative researcher and has served on a number of public health and clinical health guideline panels; QG and LP are health professionals with experience in advanced qualitative methods and public health practice, and NC is a doctoral student receiving training in quantitative and qualitative methods. This diversity of perspectives, but connection to the guideline community, means we aimed to offer a constructive critique to improve the methods of guideline development.

## Results

We approached 36 potential participants via email and interviewed 20 (10 male and 10 female). Thirteen people did not respond to emails and 3 could not commit to a time. We had a lower response rate from individuals in evidence review groups. We interviewed 16 working committee members, and 4 evidence review group members. By interview 16, similar narratives were shared. We recruited 4 additional participants representing diverse perspectives to ensure exploration of the range of experiences and determined saturation at interview 20.^20^

The experiences of the guideline development process were markedly different for the 2 key groups of participants involved in the process, the evidence review groups and the working committees. These experiences suggest that public health guideline development in Australia is a divided process. The division is driven by 3 related factors: the divergent disciplinary background and expertise that each group brings to the process; the methodological limitations of the framework, inherited from clinical medicine, that is used to assess the evidence; and barriers to communication between content experts and evidence reviewers around respective roles and methodological limitations.

### Divergent Disciplines

Participants on the working committees had divergent disciplinary backgrounds (eg, physicians, toxicologists, clinical and public health researchers) compared to those on evidence review groups (eg, systematic review experts) and held varying, sometimes conflicting beliefs about what constituted ‘good’ evidence. Many experts on the working committee viewed their primary role as protecting the public’s health through reducing possible harms to hazardous exposures or recommending interventions that would improve health outcomes. These individuals often viewed ‘good’ or ‘important’ evidence from the perspective of their own knowledge and expertise and not from the standards of methodological rigour used by the evidence review groups.

For example, one working committee member often felt that studies with statistically significant harms or benefits should be included in the body of evidence to form recommendations, even if they had been excluded by the evidence review groups on methodological grounds.

*“There was a critical study which, you know, some of the older studies were omitted because they were methodologically not considered acceptable; but, they were strong results, so it was thought they couldn’t be dismissed”* (participant #18, working committee member).

The evidence review groups, however, were contracted to evaluate the evidence using explicit methodological frameworks, such as systematic reviews and GRADE, that had very clear criteria for how the evidence was to be evaluated. The evidence review groups were aware of these disciplinary backgrounds and beliefs that the working committees brought to the guideline development and recognised the importance of having these experts involved in the process. However, they were also aware of the challenges that this presented because the evidence known and used by experts often did not meet the necessary criteria for inclusion.

*“…So you want experts, sure and of course a lot of these experts, they’re very professional and they produce really good research and of course they’re attached to that research … My experience has been a common thing when you first present the evidence review or the systematic reviews you’ve done, they get upset because it’s not what they know and it’s different – it’s looked through a different lens”* (participant #19, evidence review group member).

These divergent roles and epistemological beliefs led to conflict and division between the groups. Many of the working committee members believed that in order to best protect the public’s health, the evidence presented by the review groups should at times be challenged.

*“Yeah, it wasn’t all plain sailing, there were differences of opinion, yep, and there were differences of opinion on what should be included, because some of the people on the committee were experts in their field and had contributed to the literature, so that makes it also that little bit different”* (participant #6, working committee member chair).

The evidence reviews groups however, saw this process as being hostile, aggressive and at times they felt victimised for doing their job. They felt that the evidence-review role they had been contracted to complete was not respected by some working committee members.

*“We’re trying to do the best we can, we’re not content experts, we’re methodology experts, you know, we’re not deliberately trying to sabotage the process, we’ve got to work with people, but it always seems to come back to what feels like a very personal attack … it often is quite aggressive”* (participant #10, evidence review group member).

For at least some participants, the guideline development process was a divided one, with one dominant group offering content expertise and the other group attempting to provide methodological expertise.

### Methodological Limitations

It was widely considered by all participants that the evidence reviews, and guideline development processes should be rigorous and transparent and that this would enhance the credibility of the guidelines.

*“I think that’s NHMRCs main goal of this whole process is that it would be as transparent and reproducible as possible that every decision is documented, and process driven as much as possible but there’s a framework for each step, and I think that’s working reasonably well”* (participant #4, working committee member).

Although working committee members were generally supportive of the processes they used, they acknowledged methodological limitations. For example, several working committee members recognised that the methods used in evaluating the evidence for their public health guideline topic were designed for clinical medicine and evaluation of randomised controlled trials.

*“The problem is that NHMRC holds you to their standards of evidence, which are designed for other forms of evidence. They’re designed mainly in the medical domain and drug domain. So, to apply them to something like (topic) is ridiculous”* (participant #12, working committee member).

They described how relevant and important evidence was consistently being downgraded, leading to a body of evidence used that appeared low quality.

*“So, because it was this public health type evidence, what it meant was that none of the gradings were very high. And that we thought that – and I think many people have had the same views – that it was really not appropriate. And that the randomised clinical trial approach to evidence obviously a gold standard and so on, but that it was important not to throw out all the other things where – where randomised clinical trials were never going to be possible”* (participant #7, working committee chair).

When following GRADE guidance, little or no evidence leads to recommendations that are rated as “weak.” Several working committee participants and chairs were concerned that weak recommendations would not be understood by policy-makers in determining appropriate action to protect the public’s health or would be misrepresented by industries that may wish to discredit their findings.

*“But we had to, and we argued a lot about how to word this exactly, because if you say there’s no evidence it could mean that there just isn’t enough research to know or there is evidence that it doesn’t cause that. So we had to be very careful with the wording…So I remember we argued, and argued and argued about the wording of that to get it to a situation that we were all happy with”* (participant #16, working committee member).

However, the evidence reviews groups often felt that they, and not the methodological frameworks, were blamed for the way the evidence was evaluated.

“*I mean, that’s essentially what we did but it’s a very uncomfortable position to be in. I feel like they like the idea of it. But in practise when you give the results their sort of like, they’re shocked, and I mean, the methods can only do so much and they’re not flawless, there’s limitations they just don’t often expect what they get at the end* ” (participant #1, evidence review group member).

Despite such criticism, the evidence review groups’ members who had intimate knowledge of the methodological process and were aware of how the evidence would be presented, understood why the working committee were frustrated, and were even empathetic to these issues as they knew what the limitations of the framework were.

“*I don’t mean to be critical of the working group or the NHMRC and I think this was very challenging right from the beginning because it’s public health intervention and a good example of using the GRADE process. It’s very difficult because they’re all observational studies…and there were quite a lot of issues* ” (participant #19, evidence review group member).

### Barriers to Communication

The evidence review groups contracted by the NHMRC worked independently from the working committee for most of the process. Evidence Review group members were unable to share with the working committee insights and opinions on the best approach to identifying or evaluating the evidence. Further, the 2 groups were unable to help one another to understand each other’s point of view. The transfer of ideas, knowledge and expertise was limited by the separation of the 2 groups.

*“No, no, that’s actually a challenge too, because they decided to outsource them to a body that has expertise in doing systematic reviews, but not topic specific expertise”* (participant #5, working committee chair).

Not including content experts in the evidence review process provided the opportunity for working committees to be critical of the evaluations of the evidence review groups. For example, the content experts felt that the evidence review experts lacked the necessary knowledge on a guideline topic to identify all the relevant literature necessary to inform the guideline.

While conversely, the complexity of the methodology meant that unless working committee members had a methodological background, they found it difficult to understand and follow the evidence evaluations, when they were presented by the evidence review groups.

*“So, they would, we’d have these 2-day meetings and the people who run the tender to do the systematic review would kind of explain the methodology and, I’m not a methodologist so a lot of it went past me, about what you should include and what kind of grade recommendations could be supported, by what kind of evidence and so forth”* (participant #16, working committee member).

This highlights how by not working with the evidence review groups regularly from the outset and understanding the methods thoroughly, some working committee members felt limited in their ability to contribute to the guideline process due to their lack of methodological knowledge and training.

This separation between the groups was also seen by the evidence review groups as a major limitation in how the guideline process was conducted. The irregularity of the meetings at which the evidence reviews groups presented their findings to the working committees meant it became an ineffective way of communicating with the working committee on how the evidence was being evaluated with the methodology employed.

*“Here it would include the GRADE process, and everyone goes yeah, okay, we understand that, that’s good, but when it comes to the presentation it’s usually so long after they’ve forgotten – even for us it’s challenging”* (participant #19, evidence review group member).

As a result of this, when evidence review groups did advise the working committee on what evidence should or should not be included, they were criticised for their suggestions.

*“I don’t know. What we were told is don’t tell us what to do, which shook us quite a bit because we were like well, we’re just giving advice. Like, we don’t mind if you don’t take it but this is a wee bit challenging. So, then you don’t know what your role is* ” (participant #19, evidence review group member).

The evidence reviews groups felt that this criticism from the working committees grew from the separation of duties, and failure to have effective communication strategies in place. The evidence review groups members felt that many of the tensions that were experienced between groups of participants could have been limited if working committees were provided with clear information about the different roles of the 2 groups.

*“But certainly for these 2 NHMRC ones it felt combative and I don’t – and it’s been an unpleasant process for us and we’ve felt that either the Chair should stand up and just – it’s just little things, like just saying, you know, if someone’s attacking the work, just stop in and say, look, these guys have done and spent a lot of time and a lot of work on this so let’s just calm down and let them talk through the methodology of how they’ve done it”* (participant #10, evidence review group member).

Throughout the process evidence review groups felt a lack of support when delivering their work that was at times confronting and difficult for some working committee members to accept. Evidence review groups recognised an unequal and unfair power dynamic between the 2 groups. This led to the evidence review group members feeling that they were not valued or respected contributors to the guideline process.

*“But it’s my experience in working with these advisory committees, particularly with the NHMRC that it’s the committee that makes the decision and the evaluation group is very much, you know, in a responsive position and pretty much on the back foot”* (participant #10, evidence review group member).

While the roles and responsibilities of these 2 groups in the guideline process may not be intended to be equal, it is highlighted here that the evidence review groups felt that they played a passive role. Being in a ‘responsive position’ demonstrates how this division is perceived by the evidence review groups as a process that is dominated by a working committee that do not fully value their contribution to the guideline process.

## Discussion

The methods experts and content area experts interviewed for our study agree that rigorous methods should be used to develop public health guidelines that are considered valid and trustworthy. Our findings suggest that more attention needs to be given to the social processes influencing guideline development in order for the experts to achieve this shared goal. The division that is present in public health guideline development in Australia is driven by the divergent disciplines the 2 key groups of participants bring to the process, the methodological limitations of the framework that is used to assess the body of evidence, and the inadequate integration and clarity of the respective roles of the evidence review and working committees. These divisions were emphasised by the lack of interaction between the groups. These themes are echoed in the literature exploring the experiences of different guideline working committees using similar methodological approaches.^18,19^ Our study however, extends this prior research by not only understanding the experiences of the working committees, but also giving a voice to members of the evidence reviews groups to understand the experience from their perspective, and the social processes involved in public health guideline development.

### Strengths and Limitations

This is the first comprehensive empirical investigation, to the best of our knowledge, into the process of public health guideline development in Australia. This study included several guideline topics and participants from diverse backgrounds thus allowing us to analyse the experience of different groups of participants involved in the process.

This work reflects the opinions and experiences of the participants involved in the development of a sample of guidelines, and therefore it is possible that the experiences represented here may be different to those who did not participate. However, we sought to minimise this bias by including a diverse range of guideline topics. Four fifths of the respondents were working committee members, while only one fifth from the evidence review groups. While we attempted to contact more members of the evidence review groups, their response rate was lower and evidence review groups tend to have about half the number of members as working committees. The reason for this low response rate may be due to the evidence review groups being contracted by the NHMRC to conduct the evidence reviews and they therefore may feel conflicted in contributing as they are paid by the developer. Alternatively, they may have felt uncomfortable with sharing their thoughts and insights on the guidelines process as these experiences revealed in this study were often challenging. Future research should aim to understand these experiences further.

We feel, however, the concepts we represent here were expressed by both groups as we continued our sampling and analysis until we reached thematic saturation.^20,24^

### Our Results in Relation to Other Studies

The prioritisation of disciplinary expertise that working committee members may have over the methodological expertise typical of the evidence review groups, is consistent with previous studies that have examined how evidence is conceptualised and used in forming recommendations in public health guidelines.^17^ A previous qualitative study that explored the social processes of how evidence is understood and used by guideline advisory groups found that different group members prioritised the ‘scientific’ evidence, such as randomised controlled trials in informing their decision-making, while others their professional experiences.^17^ Clinical and practical experiences have also been shown to take precedence over the evidence in forming recommendations when using the GRADE process in the developing WHO guidelines that included public health topics.^18^ Our study expands on these studies by showing that the content and evidence review experts have differences in valuing randomised, clinical trials vs. clinical expertise and in how they value observational design studies needed for public health guideline development.

The specific methodological challenges involved with evaluating the evidence used in public health guideline development described by the participants in this study have previously been identified in a study that explored the experiences of groups that have applied GRADE for developing guidelines or systematic reviews in the field of public health.^13^ The difficulties identified included, which studies to include or exclude, the inability to upgrade the quality of evidence from observational studies higher and concerns that policy-makers may potentially misinterpret no or low quality evidence when determining what course of action to take. Further, the limited understanding of the GRADE process used to evaluate the evidence by the working committee discussed in our present study, was also demonstrated with previous investigations into the guideline development process by WHO guideline groups.^18,19^

The tensions felt by the evidence review groups with the working committee members in this present study has also been shown in a previous study that explored the experiences of methodologists working on WHO guidelines with discordant recommendations.^19^ Although methodologists were also part of the working committee in our current study, the evidence review groups were responsible for conducting and presenting the results of the initial evaluation and grading of the evidence, which makes their experiences similar to this previous investigation. Therefore, the experiences of feeling tension with the working committee members, the need for their role to be clearly articulated and the need to receive greater support from the NHMRC throughout the process, are relevant and consistent with these previous findings.^19^

## Implications for Practice, Policy and Research

### Bridging the Gap in the Divided Process

While there are methodological challenges and considerations that go beyond the scope of this paper,^12^ a number of steps could be put into place to help optimise the public health guideline development process in Australia and globally. Improvements in the social processes of guideline development could reduce the tensions and division that have been identified between the 2 key groups of participants involved in this study. Firstly, more pragmatic advice and training by the NHMRC for the working committee members unfamiliar with the frameworks used to assess the body of evidence is necessary, not only at the commencement of the process but should be ongoing thereafter. Both the working committee and evidence review groups viewed understanding the methods as a significant challenge to the current process. Inadequate understanding of the methods restricts the level of input certain working committee members can have in the process and creates tensions between the groups.^13,18,19^

Secondly, the role of the evidence review groups needs to be clearly articulated from the start and reinforced throughout guideline development process.^19^ The evidence review groups also need to feel appropriately supported in their roles when presenting the evidence reviews, particularly by the working groups chairs. Therefore, the power imbalances created between the 2 groups must be minimised through strong facilitation, which would allow for the evidence review groups to feel their contribution is respected and valued and their opinions heard throughout the process. These power imbalances could also potentially be minimised by paying attention to differences in age, experience, gender, or region between the evidence review groups and working committees.^25^

Finally, there is a need to work collaboratively from the outset and throughout the duration of the guideline process, to make it more collegial, effective and efficient. To enhance the transfer of ideas, knowledge and expertise, the physical separation that is currently present between the 2 groups should be reduced. While there may be benefits to keeping expert opinion influence away from the evidence review process, the infrequent meetings and lack of communication between the groups appears to be a significant factor in tensions that are made apparent when the evidence review groups present their findings to the working committees.^25,26^ By integrating the groups, with subgroups to evaluate the evidence for particular questions, the tensions identified from this lack of contact and communication will be significantly reduced. Furthermore, methodologists who are members of the working committees could facilitate discussions of the reviews presented by the evidence review groups. Their understanding of the challenges associated with evidence review for public health questions could bridge a gap in communication between the working committees and evidence review groups.

Through greater education of the working committee on the methodological process employed to evaluate evidence, improved communication on the role the evidence review groups play, along with better facilitation of the process so that the evidence review groups feel their contribution is respected and valued, an enhanced transfer of ideas, knowledge and expertise in the guideline development process will be possible.

## Acknowledgements

We thank the qualitative work group of the Evidence Policy and Influence Collaborative at the University of Sydney, Sydney, NSW, Australia for their involvement in discussions around the emerging concepts from the data. We also thank the staff of the Australian NHMRC for assistance with recruiting participants.

## Ethical issues

This project was approved by The University of Sydney Human Research Ethics Committee (Project number: 2017/220).

## Competing interests

Authors declare that they have no competing interests.

## Authors’ contributions

NC and LAB initiated the study. NC designed the study, conducted the interviews and prepared the original drafts of the manuscript. QG and LAB assisted with the design. QG, LAB, and LMP assisted with the data analysis. QG and LMP trained and supported NC in the data collection and analysis methods. All authors made significant contributions to the final manuscript. NC and LAB are guarantors.

## Funding

This work was supported by NHMRC project grant APP1139997. Nicholas Chartres is a recipient of the James Millner PhD Scholarship in Pharmacy from the University of Sydney, Sydney, NSW, Australia. Quinn Grundy was supported by a Postdoctoral Fellowship from the Canadian Institute of Health Research.

## Authors’ affiliations

^1^Charles Perkins Centre, Faculty of Medicine and Health, School of Pharmacy, The University of Sydney, Sydney, NSW, Australia. ^2^Faculty of Nursing, University of Toronto, Toronto, ON, Canada.

## Key messages

**Implications for policy makers**

- Improvements in the social processes of guideline development could reduce the tensions and division that have been identified between the 2 key groups of participants involved in this study – evidence evaluators and content experts.
- More pragmatic advice and training for the working committee members unfamiliar with the methodological frameworks used to assess the body of evidence is necessary.
- The evidence review groups need to feel appropriately supported in their roles when presenting the evidence reviews, particularly by the working group chairs.
- There is a need to work collaboratively from the outset and throughout the duration of the guideline process, to make it more collegial, effective and efficient.
- To enhance the transfer of ideas, knowledge and expertise, the physical separation that is currently present between the 2 groups should be reduced by integrating the groups, with subgroups to evaluate the evidence for particular questions.

**Implications for the public**

Public health guidelines are designed to protect the public’s health. Therefore, the methods and processes that are used to evaluate the evidence and formulate the recommendations need to be rigorous, transparent and free of bias. Further, the 2 groups that are responsible for developing these guidelines, the evidence review groups and working committees with content expertise, must also work effectively together. However, the current practise of these groups working separately throughout the guideline development process leads to division and conflict. The recommendations we have made in this study will lead to a more functional and unified guideline development process that best uses all the relevant expertise, and therefore may contribute to better health outcomes for the public.
